# Mapping Cancer incidence across Western Victoria: the association with age, accessibility, and socioeconomic status among men and women

**DOI:** 10.1186/s12885-019-6070-x

**Published:** 2019-09-06

**Authors:** Stephanie P. Cowdery, Muhammad A. Sajjad, Kara L. Holloway-Kew, Mohammadreza Mohebbi, Lana J. Williams, Mark A. Kotowicz, Patricia M. Livingston, Mustafa Khasraw, Sharon Hakkennes, Trisha L. Dunning, Susan Brumby, Richard S. Page, Alasdair G Sutherland, Sharon L. Brennan-Olsen, Michael Berk, David Campbell, Julie A. Pasco

**Affiliations:** 1 School of Medicine, Deakin University, Geelong, Australia; 2 Faculty of Health, Deakin University, Geelong, Australia; 3 Faculty of Health, Biostatistics Unit, Deakin University, Geelong, Australia; 4 Department of Medicine-Western Health, The University of Melbourne, St Albans, Australia; 5 University Hospital Geelong, Barwon Health, Geelong, Australia; 6 The University of Sydney, Sydney, Australia; 7 Centre for Quality and Patient Safety Research, Barwon Health Partnership, School of Nursing and Midwifery, Deakin University Geelong, Hamilton, Australia; 8 National Centre for Farmer Health, Western District Health Service, Hamilton, Australia; 9 Barwon Centre for Orthopaedic Research and Education, Barwon Health and St John of God Hospitals, Geelong, Australia; 10 South West Healthcare, Warrnambool, Australia; 11 Australian Institute for Musculoskeletal Science (AIMSS), St Albans, Australia; 12 Orygen, The National Centre of Excellence in Youth Mental Health, Centre for Youth Mental Health, St Albans, Australia; 13 Florey Institute for Neuroscience and Mental Health and the Department of Psychiatry, The University of Melbourne, Melbourne, Australia; 14 Department of Epidemiology and Preventive Health, Monash University, Melbourne, Australia

**Keywords:** Cancer incidence, Accessibility/remoteness, Socio economic status, Demographic characteristics, Age, Gender or sex, Western Victoria

## Abstract

**Background:**

Cancer is a leading burden of disease in Australia and worldwide, with incidence rates varying with age, sex and geographic location. As part of the Ageing, Chronic Disease and Injury study, we aimed to map the incidence rates of primary cancer diagnoses across western Victoria and investigate the association of age, accessibility/remoteness index of Australia (ARIA) and area-level socioeconomic status (SES) with cancer incidence.

**Methods:**

Data on cancer incidence in the study region were extracted from the Victorian Cancer Registry (VCR) for men and women aged 40+ years during 2010–2013, inclusive. The age-adjusted incidence rates (per 10,000 population/year), as well as specific incidence for breast, prostate, lung, bowel and melanoma cancers, were calculated for the entire region and for the 21 Local Government Areas (LGA) that make up the whole region. The association of aggregated age, ARIA and SES with cancer incidence rates across LGAs was determined using Poisson regression.

**Results:**

Overall, 15,120 cancer cases were identified; 8218 (54%) men and 6902 women. For men, the age-standardised rate of cancer incidence for the whole region was 182.1 per 10,000 population/year (95% CI 177.7–186.5) and for women, 162.2 (95% CI: 157.9–166.5). The incidence of cancer (overall) increased with increasing age for men and women. Geographical variations in cancer incidence were also observed across the LGAs, with differences identified between men and women. Residents of socioeconomically disadvantaged and less accessible areas had higher cancer incidence (*p* < 0.001).

**Conclusion:**

Cancer incidence rates varied by age, sex, across LGAs and with ARIA. These findings not only provide an evidence base for identifying gaps and assessing the need for services and resource allocation across this region, but also informs policy and assists health service planning and implementation of preventative intervention strategies to reduce the incidence of cancer across western Victoria. This study also provides a model for further research across other geographical locations with policy and clinical practice implications, both nationally and internationally.

## Background

Cancer is a leading burden of disease in Australia and worldwide. According to the Global Burden of Cancer 2013 report, the burden of cancer is increasing globally and has shifted from the third leading cause of death in 1990 to become the second leading cause of death behind cardiovascular disease, with over 8 million deaths caused by cancer in 2013 [1].

By 2044–2045, approximately 25% of Australian residents will be aged 65 years and over, almost double the current proportion [2]. With Australia’s ageing population rapidly increasing, the burden of chronic disease, such as cancer, and associated health service delivery and utilisation is also expected to rise [3] . Likewise, government health spending is projected to increase from 5.7% of the Gross Domestic Product in 2002–2003, to approximately 10.3% by 2044–2045 [2].

According to the Cancer Council Victoria’s ‘Cancer in Victoria: Statistics and Trends 2016 [4, 5] report, projections of cancer incidence and mortality indicate an increased burden of cancer in Victoria by 2027–2031; with the annual number of all new cancer diagnoses anticipated to increase to over 43,000 (38%) and deaths to over 13,000 (19%). When projections are analysed by sex, annual cancer diagnoses for Victorian men and women are forecast to rise 25 and 52%, respectively.

The introduction of screening programs [6, 7] has offered the opportunity to detect cancer early, resulting in increased treatment options and improved survival rates overall [5]. However, the current 5 year survival from cancer (overall) for metropolitan Melbourne residents (69%) is higher than residents from the rest of Victoria (65%) [4]. This observable difference has been attributed to reduced access to screening services in rural populations, as well as lower socio economic status (SES) [8–10] and the relocation of older individuals to more urban areas post retirement [11]. Differences in cancer incidence and survival in rural areas is a highly complex issue nationally and internationally, with factors such as ageing populations, screening and early detection, sociodemographic and tumour characteristics, treatment options and access to oncology services, all likely playing a role.

Several studies have demonstrated links between socioeconomic status and incidence of malignancies such as breast, colorectal and lung, for both men and women [12]. For instance, an American national longitudinal mortality study showed that men and women with less than high school education had increased lung cancer rate rates compared to those with college education [12]. Similarly, those with family incomes less than $12,500 had recorded lung cancer incidence rates 1.7 times higher than the incidence rates for those with incomes of greater than $50,000 [8, 12]. For Australian populations, the cross sectional study by Baade et al. concluded that women residing in the most socioeconomically disadvantaged areas of Queensland were more likely than women living in more socioeconomically advantaged areas to present with advanced breast cancer (after adjusting for individual factors such as age, occupation, marital status and indigenous status) [6, 12].

Higher rates of advanced cancers at diagnosis have been reported in those residing in more remote areas/areas of low accessibility, which in turn may explain the higher mortality rate often observed in more rural areas, despite their overall lower incidence rate [12, 13]. The systematic review by Leung et al. [9] examined existing evidence for differences in mammography screening service use between women in rural and metropolitan areas to investigate the observed lower breast cancer survival rate for women living in more remote areas. This review examined data across several countries including the United States, Korea, Croatia, Estonia, Lebanon, Northern Island and Australia. The review concluded that women living in rural areas were significantly less likely to have ever had a mammogram or an up to date mammogram. This rural disadvantage for mammography screening may contribute to the lower incidence of breast cancer and, conversely, the increased mortality among women living outside metropolitan/urban areas. The review by Jemal et al. [12] assessed cancer incidence and mortality rates for lung and bronchus, colon and rectum, female breast, prostate, stomach, liver, oesophageal and gynecological subtypes among 45 select cancer registries globally. Patterns showed that cancer rates varied by region, sex and cancer type and that overall cancer incidence rates are increasing in less developed and economically transitioning countries [10]. A systematic review on colorectal cancer in Australia by Ireland et al. [8] demonstrated that individuals with colorectal cancer (CRC) residing in regional, rural and remote areas of Australia had poorer survival rates and less optimum clinical management. They postulated that other factors such as age, SES and sex likely moderated this effect. Whilst their evidence demonstrated an overall disparity in survival for CRC, they noted that the evidence was ‘limited and somewhat inconsistent’. Thus, to further elucidate these effects on regional disparities, type of region, age and SES should be assessed among men and women to develop and implement effective interventions aimed at improving the health and welfare of regional Australians.

As cancer incidence is strongly related to age, with less than 1% of tumours occuring before age 20 and 60% of all new annual diagnoses in Australia occuring in persons older than 65 years [2, 5], it is vital to understand the impact of the increasing ageing population, as well as factors such as socioeconomic status (SES) and accessibility on cancer incidence and mortality in order to inform policy and plan improved delivery of health services. Likewise, this information will enable a profiling model for comparison to other rural regions both nationally and internationally.

This current study aimed to map the incidence rates of primary cancer diagnoses and select subtypes across the region of western Victoria by age for men and women, and to investigate the association of age, accessibility, and area-level SES with cancer incidence from 2010 to 2013.

## Methods

### Study design

**Fig. 1:**
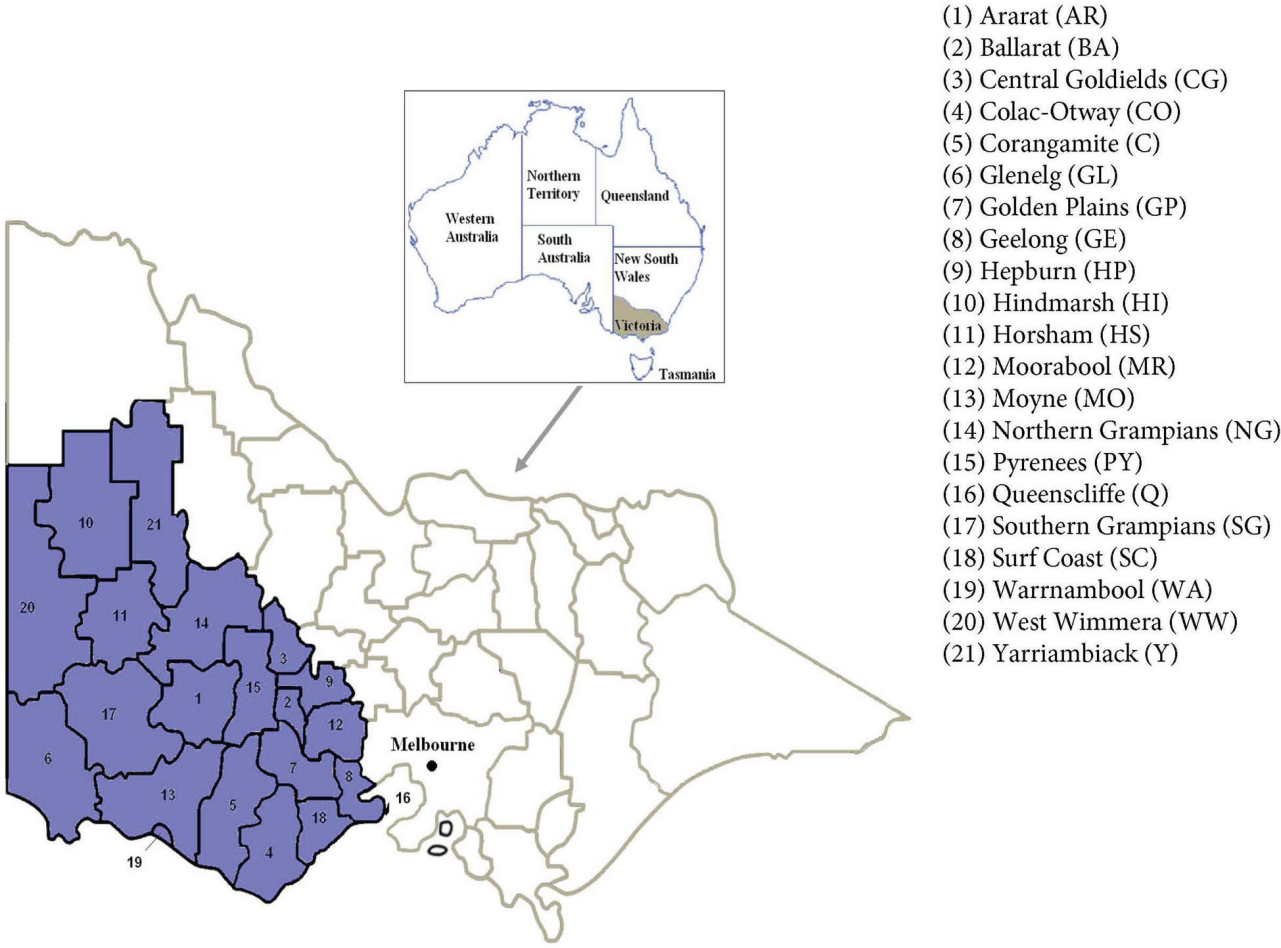
Location of the Ageing, Chronic Disease and Injury (ACDI) Study region. Local Government Areas (LGAs) included in the study are shaded. Data for graphic obtained from the Department of Health and Human Services, State Government of Victoria, Australia [14]. (Graphic prepared by MAS and KLH)

This study forms part of the Ageing, Chronic Disease and Injury Study (ACDI). Initiated in 2015, ACDI aims to provide a comprehensive snapshot of the health and wellbeing of older adults aged 40 years and over living in western Victoria, Australia (Fig. 1) [15]. The ACDI study aggregates information on demographic, socioeconomic indicators and lifestyle factors obtained from health surveys, clinical databases and government departments. Data from registers, health and emergency services, local community health centres and administrative databases are collected to generate profiles on chronic disease and injury for the study region and for sub-populations within the region.

### Study region and participants

The region of western Victoria represents close to one-third of the state by area, comprising 21 Local Government Areas (LGAs). Based on the 2011 Census of Population and Housing, the 2013 Estimated Resident Population (ERP) of the study region is 617,794, representing approximately 11% of the entire Victorian population. The three most populous LGAs of this study region are Greater Geelong (ERP = 221,515), Ballarat (ERP = 98,684) and Warrnambool (ERP = 33,300) [15].

### Data sources

The Victorian Cancer Registry (VCR) is a population-based cancer registry, which provides comprehensive information for cancer control. Notifications concerning cancer diagnoses are provided to the VCR by hospitals, pathology laboratories and cancer screening registers. The VCR records all invasive cancers, in-situ carcinomas, benign tumours and tumours of uncertain behaviour. Non-melanoma Basal and Squamous Cell skin carcinomas are not recorded. For every cancer case, demographic information such as patient name, address and date of birth, as well as tumour details including site, type, morphology, grade, behaviours and date of diagnosis are recorded [16]. Data have been collected for all cancers diagnosed in Victorian residents since 1982. Comprehensive information concerning data quality for the VCR have been provided elsewhere [17]. This most recent report included three indices of data quality; death certificate only (DCO%), histological verification (HV%) and mortality to incidence ratio (M/I%) for specific anatomical cancer sites as well as for all malignant tumours combined (2.0, 93 and 37 respectively).

Accessibility and Remoteness Index of Australia (ARIA) scores are generated by assessing distance from localities to different town categories, access to goods and services and opportunities for social interaction [18]. ARIA scores are grouped into 5 categories on a scale ranging from highly accessible (ARIA score 0.00–1.84), accessible (ARIA score > 1.84–3.51), moderately accessible (ARIA score > 3.51–5.80), remote (ARIA score > 5.80–9.08) and very remote (ARIA score > 9.08–12.00, 11]. The LGAs of Hindmarsh, West Wimmera and Yarriambiack have the highest ARIA scores (4.4, 4.1 and 3.9 respectively) in the western Victorian region and rank within the top five LGAs with the highest ARIA scores in the state, categorising them to the ‘moderately accessible’ category. These areas are largely agricultural based, predominantly producing grain and sheep [19]. The other LGAs in the study region are in the ‘highly accessible’ or ‘accessible’ categories, with no LGAs in the ‘remote’ or ‘very remote’ categories.

To assess SES, Index for Relative Socioeconomic Advantage and Disadvantage (IRSAD) scores are generated by the Australian Bureau of Statistics (ABS) using aggregated Census data for each LGA. The LGAs that were identified as being in the lowest 10% of IRSAD were the most disadvantaged, and categorised as decile 1, whilst those in the highest 10% of IRSAD were the most advantaged, and thus categorised as decile 10 [20]. With the exception of the sixth IRSAD decile, the study region encompasses LGAs across all deciles of IRSAD scores.

### Statistical analyses

Analyses for this study were performed utilising aggregated data from 2010 to 2013 inclusive, and divided into two parts: (i) the western Victorian region (ii) the 21 LGAs of the western Victorian region. Cancer incidence rate data for men and women were calculated separately, as cancer incidence differs between the sexes. For the entire study region, incidence rates were calculated separately per age group, 40–49, 50–59, 60–69, 70–79 and 80+ years for cancer [overall] and among bowel, lung, melanoma, prostate and breast subtypes. Bowel, lung, melanoma, prostate and breast cancers are the five most common cancer subtypes in Victoria, collectively accounting for 57% of all new cancers and half of all cancer deaths [5]. Data from the ABS 2011 Census Community Profile Series were utilised to undertake direct age standardisation to the 2011 Australian population [21]. Cancer incidence was expressed as Incidence per 10,000 population per year. Poisson regression was used to estimate model adjusted Incidence Rate Ratios (IRR) and their 95% confidence intervals (CIs).

For LGA level, age-adjusted incidence rates were calculated for each LGA separately. As this study utilises aggregated data on age, direct age standardisation to the 2011 Australian population was again implemented using data from the ABS 2011 Census Community Profile Series [21]. Cancer incidence rates per LGA were expressed as Incidence per 10,000 population per year and 95% CIs reported after Poisson regression analysis.

An additional analysis was conducted to investigate associations between age [age standardisation to the 2011 Australian population,] ARIA and SES (IRSAD deciles converted to quintiles) across the LGAs and corresponding cancer incidence rates. Age-adjusted incidence rates were calculated for the region and geocoding (Pitney Bowes Software Pty Ltd) performed to determine SES and ARIA codes. This analysis was performed using Poisson regression after accounting for aggregated data considering LGA as unit of analysis and incidence rate ratios (IRR) were calculated.

Minitab (version 16, Minitab, State College, PA, USA) and STATA 14 were used for analyses.

## Results

### Western Victorian region (whole study region)

**Fig. 2:**
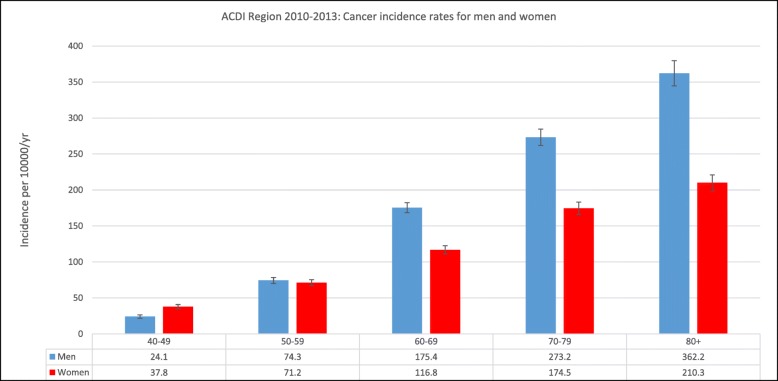
Incidence rates of all cancers for men and women in the ACDI Study region 2010–2013 inclusive. Data are presented as rates per 10,000 persons per year according to age groupings. Error bars represent 95% CIs for each age group for men and women

From 2010 to 2013 inclusive, 15,120 cancer cases (2095 bowel, 1403 lung, 1359 melanoma, 2123 prostate and 1856 breast) were identified for 8218 men and 6902 women. The incidence of cancer [overall] increased with advancing age for both men and women across the region (Fig. 2). For men, the rate ranged from 24.1 per 10,000 population/year (95% CI 21.7–26.4) in the 40–49 years age decade to 362.2 per 10,000 population/year (95% CI 344.7–379.7) in the 80+ years age group. For women, the rate ranged from 37.8 per 10,000 population/year (95% CI 34.9–40.7) in the 40–49 years age decade to 210.3 per 10,000 population/year (95% CI 199.8–220.8) in the 80+ years age group. The male incidence rate was higher than the female rate for all age groups except for 40–49 years, where the rate was 24.1 per 10,000 population/year and 37.8 per 10,000 population/year for men and women, respectively (Fig. 2).

**Fig. 3:**
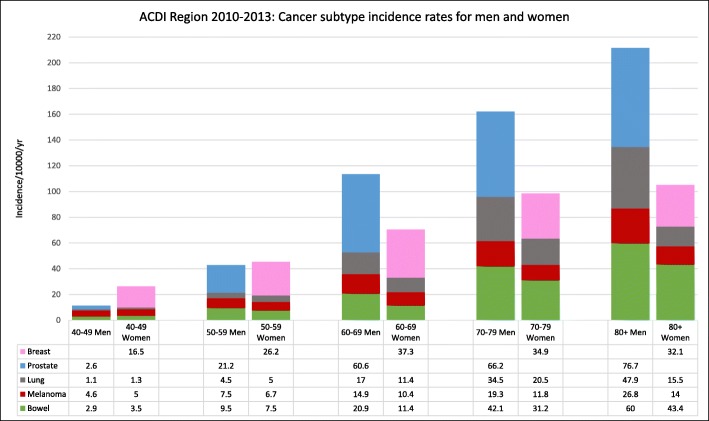
Incidence rates of colorectal, lung, melanoma, prostate and breast cancer in men and women in the ACDI Study region 2010–2013 inclusive. Data are presented as rates per 10,000 persons per year for each age grouping

Incidence rates of bowel, lung, melanoma, prostate and breast cancer in men and women in the ACDI Study region from 2010 to 2013 inclusive are shown in Fig. 3. Among each age group for women, incidence rates were highest for breast cancer. Breast cancer rates increased with increasing age from 16.5 per 10,000 population/year (95% CI 14.6–18.4) in the 40–49 age decade to 32.1 per 10,000 population/year (95% CI 28.0–36.2) in the 80+ years age group. For men, prostate cancer had the highest incidence (ranging from 2.6 per 10,000 population/year (95% CI 1.8–3.4) in the 40–49 years age decade to 76.7 per 10,000 population/year (95% CI 68.6–84.7) in the 80+ years age group in all age groups except 40–49 years where melanoma had the highest incidence rate of 4.6 per 10,000 per population/year (95% CI 3.6–5.6).

### 21 local government areas

**Fig. 4:**
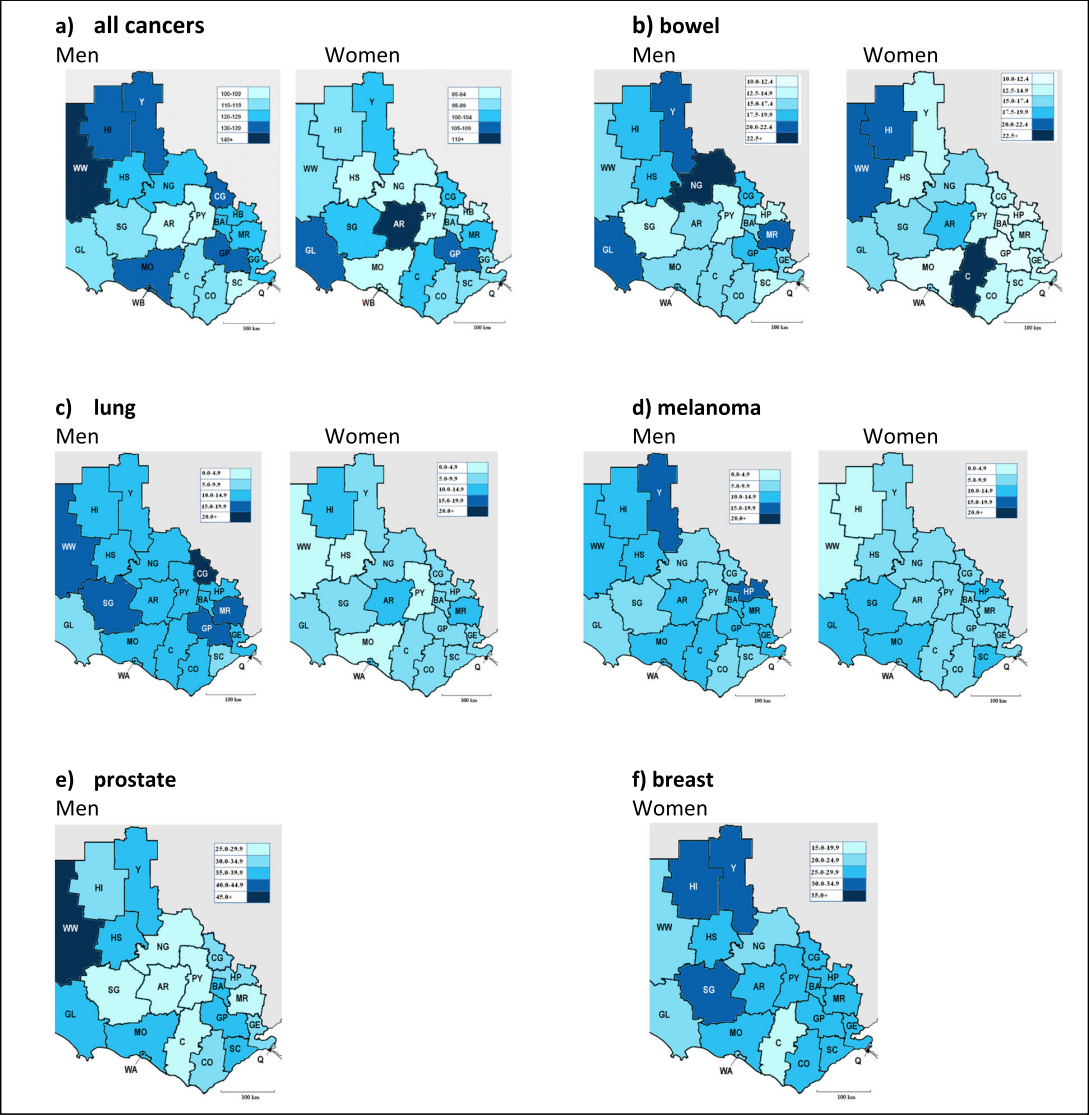
Cancer incidence rates by Local Government Area (LGA) for men and women. Configured heat maps showing age adjusted incidence rates for men and women for **a** all cancers, **b** bowel, **c** lung, **d** melanoma, **e** prostate and **f** breast cancer, aged 40+ years across the study region 2010–2013 inclusive. The legend shows the shading as incidence rate per 10,000 population/year. AR = Ararat, BA = Ballarat, CG = Central Goldfields, CO=Colac-Otway, C=Corangamite, GL = Glenelg, GP = Golden Plains, GE = Greater Geelong, HP=Hepburn, HI=Hindmarsh, HS=Horsham, MR = Moorabool, MO = Moyne, NG = Northern Grampians, PY=Pyrenees, Q = Queenscliff, SG = Southern Grampians, SC=Surf Coast, WA = Warrnambool, WW=West Wimmera and Y=Yarriambiack

Figure 4 shows data for age adjusted cancer incidence in men and women, aged 40+ years for all 21 LGAs across the study region. For men, the agriculturally based LGA of West Wimmera recorded the highest incidence rate of cancer overall (5.3 per 10,000 population/year 95%CI 4.1–6.5), and the lowest incidence rates occurred in Ararat (3.7 per 10,000 population/year 95% CI 3.0–4.3) and Pyrenees (3.5 per 10,000 population/year 95%CI 2.8–4.3). Among women, Ararat was the LGA with the highest age adjusted incidence (3.8 per 10,000 per population/year 95% CI 3.1–4.4). Heat maps displaying cancer incidence for each of the specific cancer subtypes (bowel, lung, melanoma, prostate and breast) across the study region are shown in Fig. 4.

**Fig. 5:**
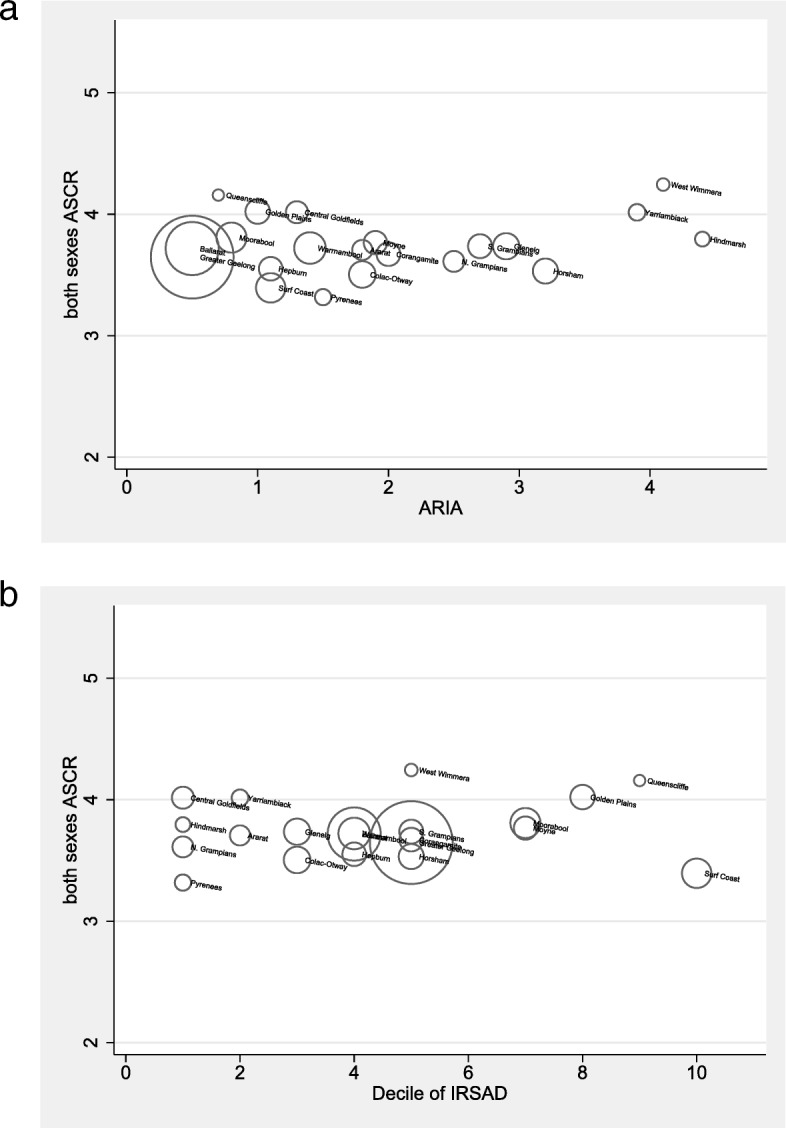
Bubble plots for association between age standardized cancer rates (ASCR), and **a** ARIA (Accessibility/Remoteness Index of Australia); **b** socioeconomic status (SES; Index for Relative Socioeconomic Advantage and Disadvantage; IRSAD) occurring during 2010–2013 (inclusive) in the region of western Victoria. Data presented for men and women are combined. LGA populations visualized in the scale of their circular bubbles. Size of bubbles indicate LGAs proportional size

After accounting for age in multivariable Poisson regression model, SES and ARIA were significantly associated with cancer incidence (all *p* < 0.001) for men and women (Table 1). Areas with greater socio-economic disadvantage (represented by lower IRSAD scores) were associated with higher cancer incidence rates. An inverse relationship was found when assessing ARIA, with those in more remote areas (represented by high ARIA scores) having an overall lower rate of cancer incidence (Fig. 5).

**Table 1:** Model adjusted Incidence Rate Ratios (IRR) for analysis of association between age standardised cancer incidence (cancer subtypes) rates for men and women from 2010 to 2013 in western Victoria and; SES (Index of Relative Socioeconomic Advantage and Disadvantage) and ARIA (Accessibility/Remoteness Index of Australia). IRRs present as mean (95% confidence interval)

SEX	Cancer Type	SES	ARIA
		Incidence Rate Ratios (95% CI)	Incidence Rate Ratios (95% CI)
MEN	Bowel	0.25 (0.18–0.35)	0.05 (0.02–0.10)
	Lung	0.21 (0.15–0.29)	0.03 (0.02–0.07)
	Melanoma	0.27 (0.20–0.37)	0.05 (0.03–0.10)
	Prostate	0.25 (0.18–0.36)	0.05 (0.02–0.10)
WOMEN	Bowel	0.27 (0.19–0.36)	0.05 (0.03–0.11)
	Lung	0.22 (0.16–0.30)	0.03 (0.02–0.06)
	Melanoma	0.16 (0.16–0.17)	0.05 (0/03–0.09)
	Breast	0.34 (0.26–0.45)	0.09 (0.05–0.16)

All *p* < 0.001*

## Discussion

This research mapped the incidence rates of primary cancer diagnoses and select subtypes across the region of western Victoria by age for both men and women, and investigated the association of age, accessibility and area-level SES on cancer incidence. In accordance with the trends in the state of Victoria, which show approximately 75% of new cancer cases in men, and 65% in women are diagnosed among those aged 60 years and over [22, 23], our study indicated that the incidence of cancer [overall] increased with increasing age for men and women across the region. Furthermore, the male incidence rate was higher than the female incidence rate for all cancers from 50 years and over, but lower for the 40–49 years age decade. This is again reflective of national figures which show significantly higher rates of cancer incidence for men than women in those aged 55 years and over and a lower incidence for men than women in those aged 30–49 years [22, 23]. The increased male incidence rate is a trend found nationally and internationally and is attributed to several key factors including the high occurrence of prostate cancer diagnosis largely found in western countries utilising PSA (prostate-specific antigen) testing [12, 23] as well as increased prevalence in men in lifestyle factors known to increase cancer risk including smoking cigarettes, increased alcohol consumption, occupational exposures, and overall poorer diet [8, 23]. The high incidence of cancer in women between the ages of 30–49 years has been largely attributed to the high incidence of breast cancer in this age group [23] and these trends were reflected in our results.

Cancer incidence rates (overall and among bowel, lung, melanoma, prostate and breast subtypes) varied between men and women across the LGAs. For men, West Wimmera recorded the highest incidence rate of cancer [overall] with the lowest incidence rates occurring in Ararat and Pyrenees., Ararat was the LGA with the highest cancer incidence rate among women. Ararat is one of the most socioeconomically disadvantaged LGAs in the western Victorian region. Low IRSAD scores indicate relatively greater socioeconomic disadvantage and a lack of advantage overall. For example, a low score would be reflective of an area with (among other factors) many households with low incomes, and/or many people in unskilled professions [24].

Results from our study further demonstrated that LGAs with greater socio-economic disadvantage, and LGAs which scored as less accessible and more remote, were associated with higher cancer incidence rates. These results correlate with the current available literature for Australian populations, which demonstrate disparities in cancer incidence between rural and metropolitan regions [5, 6, 9, 25]. Whilst disparities in incidence between metropolitan and more remote and rural areas exist, the driving forces underpinning this association are complex. Rural areas have higher levels of disadvantaged communities, are older, poorer and likely to have less access to screening and treatment services which can impact willingness and access to care [26, 27]. Rural communities may include agricultural workers, which have been shown to have increased rates of some cancers [28]. Furthermore, socioeconomic disadvantage has been associated with lifestyle factors known to directly contribute to cancer risk, such as increased levels of smoking [29], alcohol consumption, physical inactivity and poor diet [30–32]. Thus, it is likely that the observable disparity between urban/metro areas and more rural and remote areas is due to many contributing factors. Whilst these results demonstrate an association between age, sex, accessibility and SES on cancer incidence, any inference on causality would need to account for confounding variables associated with other risk factors; such as smoking, alcohol consumption, diet, physical inactivity and obesity, hormonal factors in women such as hormone replacement therapy (HRT), sunlight, radiation, occupational exposures, pollution and genetic susceptibility [22]. Likewise, stage at diagnosis may have also provided a more accurate description of the observed associations.

The ACDI study aims to describe the pattern of chronic disease and injury and its relationship with age, sex and location for the region of western Victoria. To date, this study has investigated several diseases and injuries including diabetes, fracture and joint replacement [11, 33–35]. The addition of comprehensive information regarding cancer incidence not only provides a snapshot of this disease across the region and its 21 LGAs but allows for comparison among disease and injury categories. Outcomes of this analyses can be utilised to produce a highly comprehensive community profile with the potential to improve interventions impacting cancer incidence. As Australia’s ageing population is increasing [3], so too will the burden of chronic diseases, such as cancer and other comorbidities [36]. These findings have vast implications on cancer, community and primary health services at all points of the continuum from prevention strategies, screening services, active treatment, survivorship and palliation [1]. As this study comprised a large geographic area and included populations with varying degrees of remoteness and socioeconomic advantage and disadvantage, it is uniquely posited to further raise rural and remote health disparities. An introduction to this region which further describes its novelty has been provided elsewhere [15]*.* Outcomes of this study can target healthcare utilization and management of disease locally. Importantly, this study can be utilised as a repeatable profiling model in other geographical settings, where a variety of population densities are present to identify targeted interventions to reduce disparities in cancer outcomes in regional and rural communities.

### Strengths and limitations

Mandatory notification of new cancer diagnoses is provided to the VCR by hospitals, pathology laboratories and cancer screening registers across the whole of Victoria. The VCR initiated in 1982 and is the most comprehensive and reliable cancer registry in the state, thus it is unlikely that any cases have been missed for the years (2010–2013) analysed in this study. We acknowledge that survival rates were not assessed in this study. The LGAs included in this study are highly diverse and include cities as well as regional centres and LGAs with small populations and large areas including agricultural lands. However, no LGAs in this study region were in the “remote” or “very remote” ARIA categories, and this study included the LGA of Hindmarsh, which has the highest ARIA in the state of Victoria. Thus, results may need to be interpreted with caution when addressing this model on highly remote areas. No inferences can be made at an individual level as ARIA and IRSAD were investigated on an area level, and we utilised aggregated data for analysis. Furthermore, ARIA scores do not account for access to oncology services and screening centres. Nevertheless, these data can be utilised to assist health service planning and implementation of targeted preventative and intervention strategies, screening services and treatment, survivorship and palliation procedures to ensure better service provision across western Victoria.

## Conclusion

In conclusion, results from this study identified that for the region of western Victoria, cancer incidence rates vary among men and women and across LGA and increase with advancing age, greater socio-economic disadvantage and remoteness/lower accessibility. Identifying inequalities in rural and regional health service delivery is important and it is anticipated that these findings will assist in implementing targeted and improved services at all points of the cancer continuum from prevention strategies, screening services, treatment, survivorship and palliation. This study also provides a model for further understanding of geographical locations with national and international implications.

## Data Availability

The data that support the findings of this study are available from the ACDI study, but restrictions apply to the availability of these data, which were used under license for the current study, and so are not publicly available. Data are however available from the authors upon reasonable request and with permission from the ACDI study Director (JAP).

## Abbreviations

ABS: Australian bureau of statistics
ACDI: Ageing Chronic Disease and Injury study
ARIA: Accessibility/remoteness index of Australia
BSD: Barwon statistical division
CI: Confidence Interval
CRC: Colorectal cancer
ERP: Estimated resident population
IRR: Incidence rate ratios
IRSAD: Index of relative socio-economic advantage/disadvantage
IRSD: Index of relative socio-economic disadvantage
LGA: Local government area
PHN: Primary Health Network
PSA: Prostate-specific antigen
SES: socioeconomic status
VCR: Victorian cancer registry
